# Analysis of the concept of nurses’ autonomy in intensive care units: A hybrid model* ** *** ****

**DOI:** 10.17533/udea.iee.v41n2e17

**Published:** 2023-08-30

**Authors:** Fariba Taleghani, Raziye Dehbozorgi, Monire Babashahi, Sharife Monemian, Masoume Masoumi

**Affiliations:** 1 Nursing and Midwifery School, Isfahan University of Medical Sciences, Isfahan, Iran Email: taleghani@nm.mui.ac.ir https://orcid.org/0000-0002-8266-4948 Isfahan University of Medical Sciences Isfahan University of Medical Sciences Isfahan Iran taleghani@nm.mui.ac.ir; 2 Nursing and Midwifery School, Isfahan University of Medical Sciences, Isfahan, Iran Email: rdehbozorgi110@gmail.com. Corresponding author. https://orcid.org/0000-0003-0223-4559 Isfahan University of Medical Sciences Isfahan University of Medical Science Isfahan Iran rdehbozorgi110@gmail.com; 3 Nursing and Midwifery School, Isfahan University of Medical Sciences, Isfahan, Iran Email: babashahi_m@yahoo.com https://orcid.org/0000-0002-7237-4373 Isfahan University of Medical Sciences Isfahan University of Medical Sciences Isfahan Iran babashahi_m@yahoo.com; 4 Nursing and Midwifery School, Isfahan University of Medical Sciences, Isfahan, Iran Email: monem_sh@yahoo.com https://orcid.org/ 0000-0002-2119-2617 Isfahan University of Medical Sciences Isfahan University of Medical Sciences Isfahan Iran monem_sh@yahoo.com; 5 Department of Operation Room, School of Allied Medical Sciences, Bushehr University of Medical Sciences, Bushehr, Iran Email: masoumy@yahoo.com https://orcid.org/0000-0002-9232-7064 University of Medical Sciences School of Allied Medical Sciences Bushehr University of Medical Sciences Bushehr Iran masoumy@yahoo.com

**Keywords:** professional autonomy, nurses, intensive care units, concept formation, autonomía profesional, enfermeras y enfermeros, unidades de cuidados intensivos, formación de concepto, autonomia professional, enfermeiras e enfermeiros, unidades de terapia intensiva, formação de conceito

## Abstract

**Objective:**

To analyze the concept of autonomy of nurses in Intensive Care Units (ICU).

**Methods:**

The hybrid model approach proposed by Schwartz-Barcott and Kim, which includes theoretical, fieldwork and analytical phases, was used for this study. For the theoretical and fieldwork phases, the Graneheim and Lundman stages and the CORE-Q checklist were used, and the results were combined in the final analysis phase. For the theoretical phase, 46 related articles, two instruments and four books were identified after using a search strategy in 7 bibliographic databases in English and one in Persian with the terms MESH: 'nursing', 'autonomy' and 'intensive care'. The information extracted in the theoretical phase served as the basis for the design of the questions used in the semi-structured interviews in the fieldwork phase. Eight nurses with ICU experience working in hospitals affiliated to Isfahan University of Medical Sciences (Iran) participated in the fieldwork phase.

**Results:**

The antecedents of the concept of nurse autonomy in ICUs were: empowerment of the workforce, organizational platform, and social and individual views of the profession. Its attributes were professionalism and high personal capabilities. Finally, increased personal competencies, promotion of quality of care, improved attitudes towards the profession and professional outcomes were noted as consequences.

**Conclusion:**

The autonomy of nurses in the ICU can facilitate their empowerment, which translates into the promotion of their caring behaviours, followed by the improvement of patient outcomes and quality of care.

## Resumen

**Objetivo:**

Analizar el concepto de autonomía entre las enfermeras de la Unidad de Cuidados Intensivos (UCI).

**Métodos:**

Para este estudio se utilizó el enfoque de modelo híbrido propuesto por Schwartz-Barcott y Kim que incluye las fases: teórica, de trabajo de campo y analítica. Para las fases teórica y de trabajo de campo se utilizaron las etapas de Graneheim y Lundman y la lista de comprobación CORE-Q, y los resultados se combinaron en la fase de análisis final. Para la fase teórica se identificaron 46 artículos relacionados, dos instrumentos y cuatro libros tras utilizar una estrategia de búsqueda en 7 bases de datos bibliográficas en inglés y otra en persa con los términos MESH: 'nursing', 'autonomy' e 'intensive care'. La información extraída en la fase teórica sirvió de base para el diseño de las preguntas que se utilizaron en las entrevistas semiestructuradas de la fase de trabajo de campo. En esta última fase participaron ocho enfermeras con experiencia en UCI que trabajaban en hospitales afiliados a la Universidad de Ciencias Médicas de Isfahan (Irán)**.**

**Resultados:**

Los antecedentes del concepto de autonomía de las enfermeras en las Unidades de UCI fueron: el empoderamiento de la fuerza de trabajo, la plataforma organizativa y las opiniones sociales e individuales acerca de la profesión. Sus atributos fueron el profesionalismo y las grandes capacidades personales. Por último, se señalaron como consecuencias: el incremento de las competencias personales, la promoción de la calidad de los cuidados, la mejoría de las actitudes hacia la profesión y los resultados profesionales.

**Conclusión:**

La autonomía de las enfermeras en las UCI puede facilitar su empoderamiento, lo que se traduce en la promoción de sus conductas asistenciales, seguida de la mejora de los resultados de los pacientes y de la calidad asistencial.

## Resumo

**Objetivo:**

Analisar o conceito de autonomia dos enfermeiros da Unidade de Terapia Intensiva (UTI).

**Métodos:**

Este estudo foi realizado utilizando a abordagem do modelo híbrido proposto por Schwartz- Barcott e Kim, que inclui as fases teórica, de campo e analítica. As etapas de Graneheim e Lundman e o *checklist* CORE-Q foram utilizados nas fases teórica e de trabalho de campo, e os resultados foram combinados na fase de análise final. Para a fase teórica, foram identificados 46 artigos relacionados, dois instrumentos e quatro livros após a utilização de uma estratégia de busca em 7 bases bibliográficas em inglês e outra em persa com os termos MESH: 'nursing', 'autonomy' e 'intensive care‘. As informações extraídas na fase teórica serviram de base para o desenho das questões que foram utilizadas nas entrevistas semiestruturadas da fase de trabalho de campo. Participaram desta última fase, oito enfermeiras experientes em UTI trabalhando em hospitais afiliados à Universidade de Ciências Médicas de Isfahan, no Irã.

**Resultados:**

Os antecedentes do conceito de autonomia dos enfermeiros nas Unidades de Terapia Intensiva foram: o empoderamento da força de trabalho, a plataforma organizacional e as opiniões sociais e individuais sobre a profissão. Seus atributos eram profissionalismo e grandes habilidades pessoais. Por fim, foram apontadas as seguintes consequências: o aumento das competências pessoais, a promoção da qualidade dos cuidados, a melhoria das atitudes perante a profissão e os resultados profissionais.

**Conclusão:**

A autonomia dos enfermeiros na UTI pode facilitar seu empoderamento, que se traduz na promoção de seus comportamentos assistenciais, seguida da melhoria dos resultados dos pacientes e da qualidade da assistência.

## Introduction

Autonomy plays an important role in achieving career recognition and full professional status.^1^^,^^2^. Different disciplines require different levels of autonomy; however, in any profession, there must be some degree of autonomy to enhance critical thinking and job satisfaction.^3^ In Oxford Dictionary, autonomy has been defined as the ability to operate and make decisions without being controlled by others.^4^ In Longman Dictionary, it has been defined as the ability or opportunity to make personal decisions without being controlled by another person.^5^

Despite the complexity of the issue, professional autonomy remains a topic of debate and discussion in the 21st century.^6^ Nurses’ autonomy is among the issues that have been frequently explored in numerous studies but there is a lack of understanding of the concept of autonomy in nursing literature, especially within intensive care nursing.^7^ In fact, autonomy is among the prerequisites for professionalism in the nursing profession.^8^ A nurse's autonomy is defined by Weston^9^ as “the ability of nurses to act according to their knowledge and clinical judgment which reflects and encourages the full scope of nursing practice, as defined by regulating bodies and ethical codes and values”. According to Iranmanesh *et al*.,^10^ this concept is the most important intrinsic motivation element for an occupation, which implies autonomy, responsibility, and authority and leads to the feeling of competence and belonging to a social group. 

When nurses trust their judgment and act autonomously, they will feel satisfied with their independent experience and can affect health policies. Hence, professional autonomy is considered a basic element for healthcare specialists as well as an important dimension of a healthy and positive nursing workplace. Taking care of patients is a fundamental duty of nurses as members of the healthcare team. Having meaningful autonomy would allow nurses to practice within a self-regulatory environment based on their professional judgment, make clinical decisions based on this judgment, and act accordingly within their full scope of practice.^9^ In this context, the profession encompasses control, autonomy, and the ability to use clinical decision-making and judgment for patient care. Nursing autonomy has been shown to improve patient outcomes. A nurse's sense of autonomy and accountability enables them to provide high-quality patient care, maintain patient safety, and reduce mortality.^9^^,^^12^. Intensive Care Units (ICUs) is one of the most important and highly stressful hospital wards where critically ill patients receive expensive care, and intensive care nurses often have to make urgent decisions about deteriorating patients.^13^ Nursing professionals feel an increased need for autonomy in these clinical settings and together with increased work pressure necessitate the identification of this concept. Limited autonomy in ICUs results in nurses’ restricted authority for using their personal and professional logics and moral values in patient care, eventually leading to a reduction in job satisfaction. In other words, weak nurse-physician cooperation and nurses’ low autonomy may limit ICU nurses’ ability for clinical decision-making.^14^^-^^17^

Therefore, healthcare organizations should empower and support ICU nurses to fulfill their caring responsibilities and deliver high-quality, evidence-based care to patients.^18^ Regardless, nurses discover autonomous decision-making in clinical situations to be demanding and regard the exercise of autonomy as “complex” due to the dominance of medicine, the power inequality between genders, and authoritarian leadership styles.^19^ The health care system in Iran is physician overlooked that arises from contrasts in educational experiences, the documented domination of men over women, and the historical position of physicians and nurses.^20^^,^^21^ The present study aims to analyze the concept of nurses’ autonomy in Intensive Care Units (ICUs) using the hybrid model. Schwartz-Barcott and Kim’s hybrid model combining inductive and deductive approaches have been used to clarify the basic dimensions of autonomy in ICU nurses. The strength of this method lies in the collection of data by integration of theory and practice. Therefore, it is in complete concordance with the present study objectives. 

## Methods

This study is an explanatory mixed methods study that was conducted using a sequential quantitative-qualitative design (QUAN-QUAL) consisting of three sequential steps using the approach proposed by Schwartz-Barcott and Kim’s hybrid model and also COREQ checklist^22^ and Graneheim and Lundman stages to analyze the concept of nurse autonomy in ICUs using.^23^^,^^24^ The COREQ checklist includes 32 criteria and is the only reporting guide for qualitative research using interviews and focus groups. Interviews and focus groups were designed to be reported explicitly and comprehensively via this tool.^25^ Qualitative content analysis was done using the Graneheim and Lundman stages related to the following concepts: unit of analysis, meaning unit, code, category, sub-themes, and theme.^23^ The hybrid model consisted of theoretical, fieldwork, and analytical phases, which were carried out through qualitative analysis of the phenomena while investigating texts, instruments, and articles and interviewing participants.^24^^,^^26^^,^^27^ In the theoretical phase, the concept was selected and the definitions related to autonomy among ICU nurses obtained through searching articles, instruments, and texts were compared and analyzed to achieve a comprehensive definition. During fieldwork, the setting, as well as the participants, were chosen and the data were collected and analyzed. Additionally, the differences between the definitions of ICU nurses’ autonomy and their practice in clinical settings were identified. Finally, the findings of the two aforementioned phases were compared and weighted in the analytical phase, resulting in a clear definition of nurses’ autonomy in ICUs.^27^ What follows includes the phases of analysis of the concept of ICU nurses’ autonomy in the present research.

### Phase 1: Theoretical phase

#### Search strategy for concept analysis

This phase involved a systematic approach. To understand the precise and comprehensive definition of the concept of autonomy, at first, the available books were explored, after that studies were searched via a protocol-driven strategy followed by the snowball strategy following the recommendations proposed by Greenhalgh and Peacock. Reference lists were scanned of all full-text articles and used the decision to determine whether to pursue these further Citation tracking.^28^

A procedure based on York University guidelines was designed and executed. An implementation plan included selecting review questions, including inclusion criteria, searching strategies, selecting studies, extracting data, assessing quality, synthesizing data, and disseminating conclusions.^29^ In doing so, seven English and Persian databases, namely ProQuest, PubMed, Science Direct, Scopus, Wiley, MagIran (Persian), and SID (Scientific Information Database, in Persian), were searched using ‘nursing,’ ‘autonomy,’ and ‘intensive care’ and other MESH (Medical Subject Headings).

#### Eligibility criteria

Eligible articles had the words keywords in Persian and English from April to June 2019: “nursing” OR “registered nurses” OR “personnel nurse” OR “nurse” AND “nursing autonomy” OR “autonomy” OR “professional autonomy” OR “autonomy, nursing” AND “intensive care unit” OR “unit, intensive care” OR “intensive care, unit”. in the title. Journals were searched for the period 2000 to 2019. Eligibility criteria were (1) focus on work of nurses (2) setting was ICUs (3) autonomy was discussed. At first, 2491 articles were found, which was reduced to 987 articles after eliminating the duplicates. Then, searching was filtered by language (Persian and English) and only specialized nursing journals, books, and these were selected, which decreased the number of articles to 658. Afterward, the titles and abstracts were screened for their relevance. The articles whose full texts were not available were excluded, as well. Then, the remaining full texts were evaluated for eligibility. Accordingly, the articles that showed how the concept was perceived, described, and functionalized were entered into the research. The titles and abstracts of available books and instruments also were screened for their relevance and books without full texts were excluded and extracted from the data. Finally, 46 related articles, two instruments, and four books were identified. A sample of 46 studies, 2 instruments, and 4 books was found by SHM and MM. RD, MB, SHM, and MM extracted data from each article and summarized relevant information for the review. The entire research team under the supervision of FT assessed random samples of the extracted summaries for accuracy and consistency. Disagreements between the research team were discussed.

Since this study aimed to identify the definitions, antecedents, attributes, and consequences of the concept and in order not to miss any data the credibility and quality of the texts were not evaluated. Additionally, the selection of the materials was continued until reaching a consensus regarding the depth of perceptions and explanations. Among the 46 articles obtained, 16 were qualitative, 2 were composite and 28 were quantitative and the number of participants in these 46 articles was 50,022. After investigating 46 articles, four books, and two instruments, no new information was achieved and, consequently, searching was stopped. Finally, the definitions, attributes, antecedents, and consequences were screened and integrated by the research team. 

#### Content analysis

Content analysis was used to analyze the concept of nurses’ autonomy in ICUs. The clarification process is a dimension of the content analysis strategy presented by Rogers, which includes the analysis of the existing definitions and the identification of antecedents, attributes, and consequences.^27^ In the present study, each definition was divided into meaning units and coded by two researchers. A coding table was also prepared inductively and deductively in an iterative process. In the case of the emergence of new codes, while analyzing new definitions, the table was expanded continuously. Then, it was reviewed and explored by two other researchers to ensure the extraction of the data. Finally, the codes were categorized into meaningful clusters like a powerful human workforce.

### Phase 2: Fieldwork

#### Interview with key informants

At the beginning of the fieldwork, the data extracted from the theoretical phase were used for designing questions. During the face-to-face meeting of the research team, semi-structured primary questions for the second phase of the study were written and reviewed from the data. The questions were based on PICO (Participants, Intervention, Comparison, and Outcome) and focused on the research objectives. The second phase with questions like: What is your definition of nursing autonomy in special care units? How do you evaluate the status of professional autonomy? Have there been situations in the department where you have maintained the autonomy of nursing in front of other professions, such as medicine? It was done and according to the answers of the participants, the questions continued and the interview continued until data saturation. In doing so, the data were explored by two researchers, and the questions were listed. These questions were then reviewed by two other researchers and the fieldwork was started. According to Schwartz, Barcott, and Kim, fieldwork is a basic element in concept analysis.^26^ In concept analysis using the hybrid model, qualitative data are utilized to expand insight regarding the nature of the concept. To maximize variation in responses in the present study, the samples were purposively selected from experienced ICU nurses working in the hospital's affiliated Isfahan University of Medical Sciences. The inclusion criteria of the present study were having worked in an ICU for at least a year, having at least a BSc degree, and being able and willing to express one’s viewpoints about autonomy in ICUs.

To analyze the concept of autonomy in ICU nurses, semi-structured interviews were conducted with 8 nurses with 4-13 years of work experience in the ICUs of the educational hospitals affiliated with Isfahan University of Medical Sciences. The interviews lasted for 30-60 minutes. With the permission of the participants, their voices were recorded during the interview, and notes were also taken. During the interview, the interviewer was present in the ICU as a complete observer before and after the interview, taking direct, constant observations and taking field notes. The interviews were immediately transcribed and analyzed using the approach proposed by Granheim and Landman.^23^ In so doing, the interviews were transcribed and read several times by the research team to gain an overall view. The interviews were considered the analysis unit, whole paragraphs, sentences, and even words were regarded as meaning units. There are many ways to interpret qualitative content analysis and focus on it. It is important to describe the manifest messages that each text or picture conveys, as well as the latent meanings hidden within them. The interpretation of manifest messages and latent meanings may vary in depth and level of abstraction.^23^

The research team discussed one meaning unit's latent meaning with the question: 'What does it mean on a latent level?'. The meaning units were coded based on their hidden meanings. The codes were then compared regarding their similarities and differences and were classified into more abstract categories. Finally, by comparing and carefully investigating the categories, the hidden contents of the data were introduced as the research theme. The trustworthiness of the data was assessed using the criteria proposed by Lincoln and Guba, *i.e.*, credibility, transferability, confirmability, and dependability.^30^ A prolonged engagement (5 months) was used to determine the credibility of the collected data. In addition, the research team continuously investigated and reviewed the codes, categories, and themes. Interviewees were also asked to confirm the initial codes. To determine dependability, several external observers reviewed the data analysis process, and the results were presented to the research team. The external observers were involved in the analysis and theme formation to achieve confirmability. As a final step in enhancing transferability, maximum diversity was observed in the selection of the participants.

### Phase 3: Analytical stage

Analytical reflection refers to a combination of the data obtained from the theoretical phase and fieldwork, resulting in probable changes in the definition of the concept and its filtration.^27^ In this study, the results of the qualitative data were inductively and deductively explored compared to the theoretical data using an iterative process and analyzed, the main themes were extracted, and the concept of nursing autonomy in ICU was defined based on the emerging concepts and indicators. Consequently, this final definition was supported by both theoretical and empirical data In this way, the similarities and differences between the two datasets were determined. Finally, an integrated table was prepared, which included the antecedents, attributes, and consequences obtained from articles, instruments, books, and interviews. 

Ethics approval and consent to participate. After approval of the proposal by the Ethics Committee in Biomedical Research of Isfahan university of medical sciences (IR.MUI.RESEARCH.REC.1399.252), the necessary permissions were gained from the authorities of the School of Nursing and Midwifery as well as from those of the health centers affiliated to Isfahan university of medical sciences. After introducing oneself to the unit leaders and expressing the research objectives, the researcher asked for their permission to collect data with written informed consent.

## Results

### Theoretical phase

#### Characteristics and definition of concept

Autonomy is an abstract and complex concept that nurses, having a license, gain the freedom and ability to make conscious and independent decisions based on their professional knowledge and judgment and to achieve the desired result in the scope of nursing and providing care without the permission of others. They act based on it and have control over the working condition .^1^^,^^11^^,^^15^^,^^17^^,^^31^^-^^44^ It is defined as the belief in patient-centeredness in decision-making, responsibility, and discretion, both are interdependent and the ability to perform professional work based on skill and judgment regarding patient care and clinical decision-making. It shows the freedom to make decisions based on expertise, authority, and knowledge .^45^^-^^47^ And is an important aspect of becoming a professional, which leads to the independent provider of services and plays an important role in developing the boundaries of professional autonomy.^1^ it is the ability, responsibility, and right to determine functions, implement them, and make decisions about patient needs and freedom in implementing professional values.^48^ Elsewhere, autonomy in decision-making in ethical cases related to working with patients is defined^49^ and is one of the characteristics of the nursing profession, and it is essential for quality and safe care. It also leads to autonomy in decision-making and judgment and reduces external pressure in providing nursing care.^16^

The most important factor in the state of the profession has led to its promotion and control over job performance.^7^^,^^50^ In some studies, it is more specifically related to autonomy in care, such as the ability to make decisions related to the ventilator and implement them without the direct supervision of a doctor. And also the nurse's independent decision-making based on the use of analgesic and sedation protocols in prescribing analgesics and sedation for the patient is mentioned.^51^^-^^53^ Autonomy determines the freedom of choice and makes decisions without external control^54^ It is considered social freedom and a law that makes action decisions without guidance and control outside the profession and is a rational person's capacity to make informed decisions without permission.^54^^,^^55^ The ability to guide, rethink and make decisions is a state of autonomy, accountability, authority, and responsibility in the field of doing things and the ability to act on professional knowledge in judging nursing care and clinical decisions. Autonomy has three levels: 1- Clinical Autonomy: the ability to use nursing in clinical judgment in an independent and interprofessional manner to make decisions for the patient. At this level, it is important to have knowledge and judgment about what directly relates to the patient. 2- Job autonomy: refers to operational nursing decisions that are obtained through interaction with managers in collaborative employment approaches. 3- Third, control over nursing practice refers to joint decisions that nurses make in professional practices and policies. They operate in an organization that is effective on level 2 and 3 knowledge related to the organization. ^10^^,^^46^^,^^56^^,^^57^

In another study autonomy is a human characteristic and there is a desirable quality to it, and independent action is necessary for safe and quality patient care.^17^ Professional autonomy in nursing defines the right to have any clinical and organizational judgment within the framework of a mutual health care team and by the regulations of the discipline. Professional autonomy is a dynamic process that enables the nurse to exercise independent accountability and informed decision-making in clinical judgment.^58^ In their study, Espinosa *et al*.^59^ consider the autonomy of nurses who direct palliative care. The definition of autonomy is expressed elsewhere with the concept of participatory management this approach provides an opportunity for health system employees to participate in the discussion, decision-making, and continuous improvement of work based on constant training.^60^ Critical care nurses must have the ability to make decisions about their patient's care.^61^

The results indicated that the results of fieldwork were mostly in line with those of the theoretical phase. In other words, the basic features of autonomy in ICUs were supported by the fieldwork. The only difference was related to the participants’ expression of financial motivation, which was one of the sub-subcategories of the ‘personality features’ subcategory and the ‘powerful human workforce’ category in the antecedents of the concept. Overall, reviewing the related texts and instruments, interviews, and clinical observations revealed the attributes, antecedents, and consequences for identifying the concept of autonomy in ICU nurses, which have been described in detail below. It is worth mentioning that this study aimed to explore the information about ICU nurses’ autonomy in texts, instruments, articles, and also fieldwork.

### Antecedents (based on the theoretical phase and fieldwork)

In the review of the existing studies, it was mentioned as antecedents such as knowledge *(n=15)* and skill training *(n=5)*, individual variables such as sex *(n=2)*, age *(n=4)*, education*(n=4)*, courage*(n=2)*, wishes of a person*(n=1)*, financial incentive *(n=2)*, work experience*(n=2)*, eligibility*(n=4)* and the power of independent judgment*(n=2)* and also environmental, cultural, social factors *(n=9)*, the power of the relevant organization such as freedom of action and thought *(n=3)*, overcoming the rule of medicine *(n=15)* and legal authority *(n=2)* also national laws and regulations *(n=3)*, professional leadership and management *(n=8)*, professional policy *(n=9)*, individual capabilities such as Philanthropy *(n=1)*, ethical standards *(n=2)*, responsibility *(n=4)*, accountability *(n=3)*, decision-making power *(n=16)* and communication skills *(n=3)*, and also individual characteristics in the profession *(n=13)*, professional standards *(n=2)*, professional identity development *(n=13)* and ethical principles and standards *(n=2)*. Based on the theoretical stage and fieldwork and integration of the obtained codes using an iterative process, three antecedent emerging from this concept includes a powerful human workforce, an organizational platform, and, a sociocultural platform. 

#### 1- Powerful human workforce

A powerful human workforce was one of the antecedents of the concept of ICU nurses’ autonomy, which consisted of three subcategories; i.e., demographic features, personality features, and professional competence.

*a) Demographic features.* This subcategory included such sub-subcategories as sex, age, education level, race, and job tenure: *Men are more courageous. In my opinion, entrance of the educated men into the nursing profession is highly effective. Women are calm, we always appease. If they tell me to do something tomorrow, I will do that. If they tell me that again, I will. But men are not like this* (P2).Considering the effective factors in the empowerment of the human workforce, another participant maintained: *Nurses are not independent, which can be associated with a variety of reasons, one of which being related to sex. A large number of nurses are female. They have low self-confidence or they are not interested in working autonomously or like to be dependent on someone while working. Another reason is age. Those who are younger are inexperienced and do not even think about being autonomous* (P5). The present study participants emphasized the role of demographic features such as age, sex, education level, and job tenure in ICU nurses’ autonomy. In the research carried out by Galbany-Estragues *et al*.^33^ also, participants referred to the impact of sex on nurses’ autonomy. Similarly, Katja Pursio^62^ mentioned knowledge and skills as the factors related to nurses’ professional autonomy, which represented the key role of demographic features in this concept. 

*b) Personality features.* The second subcategory of the powerful human workforce was personality features, which included the following sub-subcategories: having courage, expedition, personal desires, financial motivation -The importance of money and financial motivation for some people, which can be considered among the personality traits of each person some people considered money as an important motivating factor to increase their autonomy in their profession-, accuracy, interest in working in the ICU, correct interaction, personality growth and maturation, high-stress tolerance, humanity, moral regulations, having self-confidence, and self-esteem. Considering the need for courage as an antecedent of autonomy, one of the participants stated: *Lack of autonomy among nurses may be due to the lack of courage. For example, we have learned to say yes to everything physicians say. When a doctor comes for the clinical round, we are not able to express our opinions. Even when we know that we are right, we are afraid of making mistakes and being teased or considered a lowly educated person* (P1). Another participant emphasized the necessity for prompt decision-making in interventions:*Sometimes, we cannot wait for the resident to come due to the patient’s condition. We’re not responsible for intubation, but I saw several times that the head nurse did the intubation before the arrival of the anesthesia technician. We do this to save patients. However, if physicians come soon, they do their routine tasks* (P3). Vicki D. Lachman^63^ introduced moral courage as a prerequisite for advancement in the nursing profession. Additionally, Sung Mi-Hae^64^ conducted a study in 2011 and revealed a significant relationship between nurses’ self-confidence and professional autonomy, which confirmed the impact of personality features on ICU nurses’ autonomy. However, financial motivation was only mentioned as an antecedent in the fieldwork some nurses considered increasing salaries and benefits as one of the prerequisites for increasing autonomy in the profession: *I work in the ICU and I receive 100 tomans more than the nurse who works in other wards. This difference does not motivate me to work autonomously. If the payment is increased, we will work more efficiently* (P3); *The nurses who work in private cardiac ICUs receive more. Besides, the system wants them to do a series of tasks, which leads them to feel more autonomous* (P5). Ruth McDonald^65^ disclosed the impact of financial motivation on the quality of care provided by nurses and physicians, but not on nurses’ professional autonomy. In another research performed by Baljoon^66^ in 2018, autonomy was found to be a factor in increasing motivation and decreasing job quit amongst nurses. In other words, autonomy was mentioned as a factor for the continuation of working in clinical settings, which was the contrary to the results obtained in the present investigation.

*c) Having professional competence.* Having professional competence based on the theoretical phase and fieldwork was the last subcategory of a powerful human workforce, which included knowledge and performance competence, strong clinical reasoning, competence and skills, professional specialty and skills, ability to judge autonomously, unlimited use of one’s knowledge and skills, responsibility and accountability, decision-making capability, building relationships(The ability to establish proper communication with other professions in the health system), problem-solving ability, perceived strength in clinical centers, the necessity to make decisions and act quickly depending on patients’ conditions, interdisciplinary performance, maintenance of professional autonomy in teamwork, having authority for self-assessment, differentiation, and ability to apply knowledge. All these capabilities obtained from phase one were also confirmed by phase two and are among the prerequisites of a nurse for independent performance in the health system. One of the study participants discussed the unlimited utilization of knowledge and skills, having the ability to make decisions, expedition, and knowledge competence: *In my opinion, autonomy implies that nurses have freedom of action and take responsibility for their activities. It means that nurses make decisions based on the knowledge they have gained about their profession without worrying about the occurrence of legal problems* (P1). Another participant considered the ability to forge effective relationships with colleagues as an influential factor in the maintenance of autonomy: *I have seen that the nurses who have better relationships with physicians are more trusted by the system, of course, if they have the required knowledge* (P1). It was also found that quick clinical decision-making increased nurses’ autonomy in ICUs: *Patients in ICUs are in worse conditions. Therefore, they need more autonomous, prompt decisions. Besides, the devices are complicated and nurses need some levels of autonomy to work with them* (P7). The results also indicated that having a specific job description and acting accordingly would lead to professional competence: *Some duties are mixed up. Nurses do some tasks due to their work conscience, but they will not be able to carry out their responsibilities. Thus, they become tired and feel that they have to do everything or they have to do the tasks that other people don’t do. There is no clear job description and nurses have to do what they are not responsible for* (P2). The necessity of teamwork was yet another subcategory of professional competence. *This is our fault most of the time. We don’t believe in ourselves, we don’t do what we know is right, and we cause a challenge for each other* (P8). Particular specialties and skills were also found to enhance professional autonomy: *The more specialist nurses such as respiratory nurses, wound specialists, ICU nurses, and gastroenterology nurses, the higher the professional autonomy will be* (P2). Considering professional responsibility as a subcategory of professional competence, one of the participants said: *Nurse should be aware that the patient’s life is in their hands. They shouldn’t say that care is useless and the patient is dead. I remember a man who was admitted to our ward. His consciousness level was 3, which reached 5 and he left here. Five months later, he came to the ward and said that he remembered our voices* (P3). Based on the present study findings, ICU nurses have to strengthen their professional competence to achieve professional autonomy. In the same line, Weston *et al.*^67^ emphasized the necessity for increasing nurses’ clinical competence and developing their decision-making skills to promote professional autonomy. Katja Pursio^62^ also showed the necessity of nurses’ competencies for achieving autonomy.

#### 2-Organizational platform

Considering the antecedents of autonomy among ICU nurses, the second theme was organizational platform that referred to an organizational regulations and organizational culture.

*a) Organizational regulations.* Organizational regulations included professional support, liability insurance, legal authority, acceptance of nurses’ autonomy by insurance companies, the opportunity for autonomous decision-making and function, freedom of action and thought, the new job description for nurses, tariff setting for nursing services, limited payback, cooperation in policymaking and rule setting, institute’s policies, organizational and national laws, legal identification of professional performance boundaries, legal license for autonomy, existence and application of care scales and protocols, and sufficient equipment. Regarding freedom of action and legal authority, one of the participants maintained: When laws are adopted by policymakers that support the autonomy of nurses, then nurses will work independently without fear of being called to account (P6). Professional support was also found to enhance ICU nurses’ autonomy: *Fear from the occurrence of legal problems may be a reason for the reduction of autonomy…I do my job accurately, but how much can I count on the head nurse or the matron? How much support will they provide? Will their support be effective?* (P7).In terms of tariff setting for nursing services, one of the participants said: *When we don’t receive money for the tasks we do, we will not be autonomous* (P2); *Medical dominance in clinical settings is highly effective in nurses’ autonomy. For instance, the hospital manager is a physician. Everything has been defined for physicians…Physicians are even paid for some procedures that have been done by nurses. Under these circumstances, physicians do not let us work autonomously. If financial issues were not a problem, nurses would be paid for what they did, which could consequently enhance their autonomy* (P4); *Accreditation of nurses requires a defensive force* (P6).

*b) Organizational culture.* The subcategories of organizational culture were overcoming medical sovereignty, leadership style, nursing managers’ behaviors, group adaptability, physicians’ view towards nurses’ autonomy, physicians’ trust in nurses, reduction of physicians’ monitoring, overcoming medical hegemony, the existence of strong managers in the nursing profession, autonomous leadership and management, giving some managerial authorities to nurses without the interference of other treatment team members, defending nurses’ proper performance on the part of nursing managers in front of physicians, and other healthcare teams’ trust in nurses. Managers’ power was also reported to increase support for nurses, thereby enhancing autonomy in this profession. *More powerful authorities may provide nurses with more support…* (P2). In line with the present study, Ulrich revealed the direct impact of organizational factors and regulations on nurses’ autonomy.^68^

#### 3- Society’s sociocultural platform

This category included social and individual views towards the profession, equity among the treatment team members, valuing autonomous performance, workplace (urban/rural, clinic/hospital), and cultural, social, political, economic, religious, and traditional factors.Considering the effect of the workplace on nurses’ autonomy, one of the participants maintained: *Nurses sometimes take tests for each other. They may not have the sufficient motivation or the hospital environment may have convinced them that there is no difference between having and not having knowledge* (P1). Regarding the social view and impact of culture, one of the participants said: *Our major was long among the low-level occupations. Of course, people have a better view of the profession nowadays, but they still consider us as mere service providers. Nothing more is expected from us and, as a result, we don’t try to be autonomous* (P6). In the present study, the nurses discussed the negative effect of culture on autonomy. In contrast, Ingrid Hanssen^69^ mentioned autonomy and freedom as the inseparable elements of reasoning as well as the natural components of maturity in western culture. In other words, the ideal western autonomy is a part of the cultural heritage. Regarding the lack of equity between nurses and physicians, one of the study participants mentioned: *We are not independent. There is no equity between us and physicians. If we were considered at an equal level to physicians and were valued as much, we could make decisions more easily and work autonomously* (P4). The above mentioned participant referred to the lack of equity between physicians and nurses as a factor preventing nurses from achieving professional autonomy. Consistently, Evanthia Georgiou^48^ conducted a study in Cyprus and reported a low level of cooperation between nurses and physicians in terms of patient care as well as a moderate level of autonomy amongst nurses. Furthermore, Daniel Salhani^70^ pointed to the negative effects of political, economic, religious, and traditional factors, but none of the participants mentioned these factors in clinical settings.

### Attributes (based on the theoretical phase and fieldwork)

Nurses in special care units must have individual skills such as the ability to make clinical decisions about their patient's care^61^. In various articles, it was mentioned to have individual characteristics such as professional autonomy *(n=8)*, independent decision-making and performance *(n=20)*, competence *(n=1)*, professional skill *(n=6)*, professional performance *(n=9)*, scientific performance and awareness of the field of action *(n=7)*. The evolution of the role in the profession was also mentioned in various articles by the ability of the individual *(n=6)*, the promotion of critical thinking *(n=2)*, and the acceptance of high levels of responsibility *(n=2)*. Adherence to the profession was also mentioned in the studies in the form of paying attention to accountability *(n=4)*, commitment *(n=3)*, planning *(n=2)*, and increasing the levels of empowerment *(n=3)*. Adherence to ethical and valuable criteria *(n=5)* was also mentioned in some studies. Based on the theoretical phase and fieldwork, two main themes; i.e., professionalism and personal capabilities were the attributes of the concept of autonomy in ICU nurses. 

#### 1- Professionalism

The subcategories of this attribute were professional autonomy, professional skills, scientific performance, knowledge, value, commitment, accountability for one’s responsibilities, adherence to moral issues, legal privileges, and controlling adherence to the regulations of the profession. Concerning the importance of professional knowledge and attitude and the need for deep professional knowledge in this theme, one of the participants stated: *From my perspective, the most important point is that we should learn and believe in our lessons. Sometimes, nurses have learned something, but they don’t believe in it or they may have memorized the lesson…* (P2). Similarly, Marla J. Weston and Gail Holland Wade^67^^,^^71^ revealed the necessity of educational and skill competencies in nurses, which led to their professional autonomy. Accountability for one’s responsibilities was yet another category extracted from professionalism: *If the physicians did the right task and received income and I did the right task and received income, they would be responsible for their tasks and I would be responsible for mine* (P2). Gilmore, as cited by Nouri,^39^ also emphasized autonomy alongside accountability as the prerequisite for professional nursing performance. 

#### 2- Personal capabilities

This theme involved critical thinking, responsibility, decision-making, and autonomous performance. One of the participants believed that a lack of decision-making and independent performance would be accompanied by a lack of autonomy: *When I work in a place where I know that I have some authority and I don’t have to obey others, I will have a higher level of motivation, and I will feel more responsible, I will try to keep up-to-date because I know that I have to make decisions. However, when the physician is the one who makes decisions, I say to myself that we will do whatever the physician says in case of problems; the physician is responsible in any event* (P2): *Nurses should make decisions for patients irrespective of the routines and physicians’ orders. They should provide patients with the best healthcare depending on the conditions and take responsibility for what they have done. They should do this according to the knowledge they have gained* (P8). The present study findings revealed responsibility as one of the attributes of autonomy amongst ICU nurses. Katerina^19^ also disclosed that a high level of accountability, responsibility, and autonomy was required in ICUs to optimize patients’ outcomes.

### Consequences (based on the theoretical phase and fieldwork)

The consequences of the concept of nurses' autonomy have been mentioned in many articles: professionalization *(n=17)*, nurses' autonomy *(n=9)*, improvement of patient outcomes *(n=15)*, improvement of nurse outcomes *(n=8)*, improvement of organizational outcomes *(n=12)*, promotion of individual performance *(n=14)*, promotion of organizational performance *(n=12)*, increasing value *(n=7)*, increasing satisfaction *(n=21)* and adherence to moral principles *(n=5).* Based on the theoretical stage and fieldwork, four main themes were obtained regarding the consequences of ICU nurses’ autonomy.

#### 1- Increased personal competency

The consequences of autonomy in ICUs included increased responsibility, credit, motivation to continue education, implementation of creative ideas, the performance of research activities, promotion of clinical judgment, and critical thinking. In this regard, one of the participants stated: *If we can act autonomously, we will have a higher level of motivation to improve our information and even continue our education, because we know that we will be able to act autonomously in case of having a higher level of knowledge* (P6). Increased motivation for continuing education and working in the profession has also been expressed in the book titled Autonomy and Empowerment of Advanced Practice Nurses in New Mexico as well as in the study carried out by Riitta-Liisa Lakanmaa.^7^^,^^54^ Polly ^et al^. also conducted a study in 2017 and indicated individual capabilities as a consequence of nurses’ autonomy.^72^ Similarly, Motamed-Jahromi^34^ demonstrated that increased responsibility was one of the consequences of nurses’ autonomy. Increased decision-making power and critical thinking were other consequences mentioned by Stewart in 2004.^73^

#### 2- Promotion of care quality

Autonomy was found to enhance the quality of patient care. In this regard, one of the participants said: *If we are autonomous, we have our care protocols and we know what to do with patients without waiting for the physician. This is good for patient safety, as well* (P2). Promoted care quality was one of the basic consequences of autonomy among ICU nurses, which has been confirmed in numerous studies.^19^^,^^34^^,^^43^^,^^54^^,^^71^^,^^73^^,^^74^ Moreover, autonomy was found to reduce costs as well as the length of hospital stay: *Hospital-acquired infections will decrease and lower costs will be imposed on patients. It will also be beneficial for patients in terms of safety. In my opinion, it will be most beneficial for patients* (P6); *Experienced individuals do many tasks independently. They do something, which is exactly ordered by physicians. This accelerates the process of patient care. Overall, it increases patient safety and accelerates the care process* (P3). Reduction of the length of hospital stay and costs was another important consequence, which was mentioned by Polly in 2012, as well.^54^

#### 3- Improvement of the view towards the profession

In this respect, one of the participants maintained: *It is important to have approved protocols. I sometimes feel that even the protocols coming from the Treatment Deputy are old and that is why physicians do not accept them. If they know that our protocols are up-to-date, they will accept them to be used in clinical settings, which will be effective in improving the view towards the nursing profession* (P2). Many researchers have also argued that professionalism, specialism, and socialism could promote the view toward the nursing profession.^17^^,^^37^^,^^45^

#### 4- Organizational consequences

This theme included the facilitation of healthcare provision, increased adherence to guidelines and protocols, increased knowledge-based performance, and effective leadership. In terms of knowledge-based performance and adherence to protocols, one of the study participants said: *If nurses are autonomous, they will be motivated to perform more efficiently based on protocols. In this way, they will try to learn accurately and will be able to provide more professional care services* (P7). In agreement with the present study findings, Nouri, Tao, and Carolyn Elaine Disher^15^^,^^53^^,^^74^ indicated that commitment to the profession and the organization resulted in higher adherence to regulations, as a consequence of nurses’ autonomy. Tume^51^ also reported the increased adherence to guidelines and protocols as an important consequence of nurses’ autonomy. Increased knowledge and experience was yet another organizational consequence disclosed by Baykara in Turkey.^1^ On the other hand, Panunto introduced a lack of autonomy as a factor in nurses’ non-adherence to the profession.^50^

### Analytical reflection (based on the analytical phase)

A comparison of the concept of autonomy in the articles to that described by the key informants in the experimental phase indicated that the only difference between the data obtained from the fieldwork and the theoretical phase was related to financial motivation. This was related to the personality features, as one of the attributes, was mentioned in the clinical setting, but was not found to be among the antecedents of nurses’ autonomy in the explored articles and texts. Furthermore, the negative effects of political, economic, religious, and traditional factors related to the society’s sociocultural platform were among the antecedents expressed in the articles, while they were not emphasized in the clinical setting. The integrated overview of the antecedents, attributes, and consequences of this concept has been presented in Table 1.

**Table1. The:** integrated view of the antecedents, attributes, and consequences of the concept

Antecedents	Attributes	Consequences
1- Powerful human workforce	1- Professionalism	1- Increase of personal competencies
a) Demographic features	2- Personal capability	2- Promotion of care quality
b) Mental and personality features		3- Improvement of attitude towards the profession
c) Having professional competence		4- Professional outcomes
2- Organizational platform		
3- Sociocultural platform		
Definition: According to the results of the present study, “autonomy of nurses in intensive care units” has antecedents such as powerful human workforce, organizational platform, and sociocultural platform with the attributes of professionalism and personal capability, and can cause increase of personal competencies, promotion of care quality, improvement of attitude towards the profession, and professional outcomes.		

## Discussion

The present study findings provided an overview of the concept of nurses’ autonomy in ICUs after reviewing texts, articles, instruments, and nurses’ perceptions. Clarification of the attributes of nurses’ autonomy in ICUs can help develop this concept in the health systems and promote nurses’ professional identity.^75^ In the current research, the antecedents of the concept of nurses’ autonomy in ICUs were the powerful human workforce, organizational platform, and society’s sociocultural platform. Based on the results, mental and personality characteristics, and professional competence could result in having a powerful human workforce, thereby promoting autonomy among ICU nurses. In the same line, Schutzenhofer^76^ revealed a significant relationship between nurses’ autonomy and nursing education, clinical specialty, functional role, membership in professional organizations, gender stereotypes, and personality. Sung *et al.*^64^ also showed that mental and personality characteristics such as professional self-concept and self-esteem were positively correlated to professional autonomy and job satisfaction amongst nurses. In the research carried out by Iliopoulou *et al.,*^19^ young nurses presented a lower level of autonomy. Additionally, female nurses were more autonomous, which was contrary to the present study findings. Labrague *et al*.^11^ also performed a study in 2019 and demonstrated that age, work experience, and education level were effective variables in nurses’ autonomy. Accordingly, experienced nurses gained higher scores of autonomy. In the research performed by Amini et al. in 2015,^77^ male nurses and those aged 30-40 years showed considerably higher autonomy compared to females and other age groups.

Another antecedent of autonomy was professional competence, which involved the ability to build effective relationships with other treatment team members. Similarly, Maylone *et al*.^78^stated that teamwork and cooperation between nurses and physicians were necessary to reach professional autonomy, which could eventually strengthen patients’ outcomes, increase their safety, and promote care quality. In addition, having sufficient knowledge and technical experience were among the prerequisites for professional autonomy, as mentioned in the nurses’ autonomy instrument.^79^ The other antecedents obtained in the current study were organizational platforms and sociocultural platforms. Considering the diversity of cultures around the world, Kuwano *et al.*^80^ argued that the incorporation of transcultural nursing content in educational curricula in universities and hospitals could enhance cross-cultural sensitivity and improve nurses’ professional autonomy. Generally, organizational variables such as assigning a large number of patients to each nurse, a variety of hospitals, a shortage of staff, and organizational policies and regulations can restrict nurses’ autonomy.^11^ The organizational platform was also one of the antecedents of this concept in the current investigation. Consistently, Allahbakhshian *et al.*^61^ indicated that nurses encountered two main barriers to achieving professional competence: profession-related and organization-related barriers. The obstacles related to the profession included the inability to apply professional autonomy and the lack of professional nursing organizations such as nursing associations for professionally directing the nursing profession. The organizational barriers included role conflicts, unsupported workplaces, and lack of support and encouragement on the part of managers.

In the current study, the attributes of the concept of autonomy in ICU nurses were professionalism and individual capabilities such as critical thinking, responsibility, decision-making, and autonomous performance. Considering professionalism, Iliopoulou^19^ stated that education, role empowerment, and support were required for ICU nurses to achieve their maximum professional potential. In the present study also, professional competence including the need for increased knowledge and skills was one of the important antecedents. The consequences obtained from theoretical and practical concept analysis in the present research included increased personal competence, improved care quality, organizational consequences, and professional consequences. In the same line, Labrague^11^ showed the positive impact of autonomy on organizational commitment, job satisfaction, and performance. Overall, the combination of the data obtained from the theoretical phase and fieldwork and their analysis resulted in the emergence of three main categories, namely antecedents, attributes, and consequences, of autonomy among ICU nurses, which can be presented to health managers and policymakers.

## Conclusion

This study aimed to analyze the concept of nurses’ autonomy in ICUs by describing its antecedents (powerful human workforce, organizational platform, and society’s sociocultural platform), attributes (professionalism and personal capabilities), and consequences (increased personal competence, improved care quality, improved view towards the profession, and improved outcomes) using a hybrid model.

## References

[1] Baykara ZG, Şahinoğlu S (2012). An evaluation of nurses’ professional autonomy in Turkey. Nurs. Ethics..

[2] Traynor M, Boland M, Buus N (2010). Professional autonomy in 21st century healthcare: Nurses’ accounts of clinical decision-making. Soc. Sci. Med..

[3] Mastekaasa A. (2011). How important is autonomy to professional workers?. Prof. Prof..

[4] Stevenson A. (2011). Oxford dictionary of English.

[5] Richards JC, Schmidt RW (2010). Longman dictionary of language teaching and applied linguistics.

[6] Gagnon L, Bakker D, Montgomery P, Palkovits JA (2010). Nurse autonomy in cancer care. Cancer Nursi..

[7] Lakanmaa R-L, Suominen T, Ritmala-Castrén M, Vahlberg T, Leino-Kilpi H (2015). Basic competence of intensive care unit nurses: cross-sectional survey study. BioMed Res. Int..

[8] Levine DLS (2020). Faculty Descriptions of Teaching Strategies for Professionalism in Nursing Students.

[9] Weston MJ (2008). Defining control over nursing practice and autonomy. J. Nurs. Adm..

[10] Iranmanesh S, Razban F, Nejad AT, Ghazanfari Z (2014). Nurses’ professional autonomy and attitudes toward caring for dying patients in South-East Iran. Int. J. Palliat. Nurs..

[11] Labrague LJ, McEnroe‐Petitte DM, Tsaras K (2019). Predictors and outcomes of nurse professional autonomy: A cross‐sectional study. Int. J. Nurs. Pract..

[12] Keith L, Cianelli R (2014). Exploring the concept of nurse engagement related to patient experience. Horiz. Enferm..

[13] Hartog CS, Benbenishty J (2015). Understanding nurse-physician conflicts in the ICU. Intensive Care Med..

[14] van Dam K, Meewis M, van der Heijden BI (2013). Securing intensive care: towards a better understanding of intensive care nurses’ perceived work pressure and turnover intention. J. Adv. Nurs..

[15] Tao H, Ellenbecker CH, Wang Y, Li Y (2015). Examining perception of job satisfaction and intention to leave among ICU nurses in China. Int. J. Nurs. Sci..

[16] Sarkoohijabalbarezi Z, Ghodousi A, Davaridolatabadi E (2017). The relationship between professional autonomy and moral distress among nurses working in children's units and pediatric intensive care wards. Int. J. Nurs. Sci..

[17] Paganini MC, Bousso RS (2015). Nurses’ autonomy in end-of-life situations in intensive care units. Nursi. Ethics..

[18] Mrayyan MT. (2004). Nurses’ autonomy: influence of nurse managers’ actions. J. Adv. Nurs..

[19] Iliopoulou KK, While AE (2010). Professional autonomy and job satisfaction: survey of critical care nurses in mainland Greece. Journal of advanced nursing. J. Adv. Nurs..

[20] Asad ZM, Ebadi A, Karami ZA, Gholami M, Farsi Z (2007). The relationship between nurse's perception of their head nurses empowerment behaviors and their own work effectiveness.

[21] Nasrabadi AN, Emami A (2006). Perceptions of nursing practice in Iran. Nurs. Outlook..

[22] EQUATOR Enhancing the QUAlity and Transparency Of health Research UK: The UK EQUATOR Centre.

[23] Graneheim UH, Lundman B (2004). Qualitative content analysis in nursing research: concepts, procedures and measures to achieve trustworthiness. Nurs. Educ. Today..

[24] Schwartz‐Barcott D, Patterson BJ, Lusardi P, Farmer BC (2002). From practice to theory: tightening the link via three fieldwork strategies. J. Adv. Nurs..

[25] Tong A, Sainsbury P, Craig J (2007). Consolidated criteria for reporting qualitative research (COREQ): a 32-item checklist for interviews and focus groups. Int. J. Qual. Health Care..

[26] Schwartz-Barcott D, Kim HS, Rodgers BL, Knafl KA (2000). Concept Development in Nursing Foundations, Techniques, and Applications.

[27] Rodgers BL, Knafl KA (2000). Concept development in nursing: Foundations, techniques, and applications.

[28] Greenhalgh T, Peacock R (2005). Effectiveness and efficiency of search methods in systematic reviews of complex evidence: audit of primary sources. Bmj..

[29] Akers J, Aguiar-Ibáñez R, Baba-Akbari A (2009). Systematic reviews: CRD’s guidance for undertaking reviews in health care.

[30] Polit DF, Beck CT (2004). Nursing research: Principles and methods.

[31] Setoodegan E, Gholamzadeh S, Rakhshan M, Peiravi H (2019). Nurses’ lived experiences of professional autonomy in Iran. Int. J. Nurs. Sci..

[32] Dorgham SR, Al-Mahmoud S (2013). Leadership styles and clinical decision making autonomy among critical care nurses: A comparative study. J. Nurs. Health Sci..

[33] Galbany-Estragués P, Comas-d’Argemir D (2017). Care, autonomy, and gender in nursing practice: A historical study of nurses’ experiences. J. Nurs. Res.

[34] Motamed-Jahromi M, Jalali T, Eshghi F, Zaher H, Dehghani SL (2015). Evaluation of professional autonomy and the association with individual factors among nurses in the Southeast of Iran. J. Nurs. Midwifery Sci..

[35] Shohani M, Rasouli M, Sahebi A (2018). The Level of Professional Autonomy in Iranian Nurses. J. Clin. Diag. Res..

[36] Papathanassoglou ED, Karanikola MN, Kalafati M, Giannakopoulou M, Lemonidou C, Albarran JW (2012). Professional autonomy, collaboration with physicians, and moral distress among European intensive care nurses. Am. J. Crit. Care..

[37] Varjus S-L, Suominen T, Leino-Kilpi H (2003). Autonomy among intensive care nurses in Finland. Intensive Crit. Care Nurs..

[38] Skår R. (2010). The meaning of autonomy in nursing practice. J. Clin. Nurs..

[39] Nouri A, Jouybari L, Sanagoo A (2017). Nurses’perception of factors influencing professional autonomy in nursing: a qualitative study. Nurs. Midwifery J..

[40] Petersen PA, Way SM (2017). The role of physician oversight on advanced practice nurses’ professional autonomy and empowerment. J. Am. Association of Nurse Practitioners.

[41] O’neill CS, Yaqoob M, Faraj S, O’neill CL (2017). Nurses’ care practices at the end of life in intensive care units in Bahrain. Nurs. Ethics..

[42] Maharmeh M. (2017). Understanding critical care nurses’ autonomy in Jordan. Leadershn Health Serv..

[43] Lewis FM, Soule E (2006). Autonomy in nursing. Ishikawa J. Nurs..

[44] Abril FGM, Vanegas JCR (2014). Validez y confiabilidad de la Escala de Autonomía del Índice de Características del trabajo de Enfermería (SNJCI) en Bogotá-Colombia. Enferm. Glob..

[45] Papathanassoglou ED, Tseroni M, Karydaki A, Vazaiou G, Kassikou J, Lavdaniti M (2005). Practice and clinical decision‐making autonomy among Hellenic critical care nurses. J. Nurs. Manag..

[46] Rao AD, Kumar A, McHugh M (2017). Better nurse autonomy decreases the odds of 30‐day mortality and failure to rescue. J. Nurs. Scholarsh..

[47] Petersen PA, Keller T, Way SM, Borges WJ (2015). Autonomy and empowerment in advanced practice registered nurses: Lessons from New Mexico. J. Am. Assoc. Nurse Pract..

[48] Georgiou E, Papathanassoglou ED, Pavlakis A (2017). Nurse‐physician collaboration and associations with perceived autonomy in Cypriot critical care nurses. Nurs. Crit. Care..

[49] Karanikola MN, Albarran JW, Drigo E, Giannakopoulou M, Kalafati M, Mpouzika M (2014). J. Nurs. Manag..

[50] Panunto MR, EdB Guirardello (2013). Professional nursing practice: environment and emotional exhaustion among intensive care nurses. Rev. Lat. Am Enfermagem..

[51] Tume LN, Kneyber MC, Blackwood B, Rose L (2017). Mechanical ventilation, weaning practices, and decision making in European PICUs. Pediatri. Crit. Care Med..

[52] Sneyers B, Laterre P-F, Perreault MM, Wouters D, Spinewine A (2014). Current practices and barriers impairing physicians’ and nurses’ adherence to analgo-sedation recommendations in the intensive care unit-a national survey. Crit. Care..

[53] Disher CE. (2012). Registered nurses' access to autonomous self-governance structures and retention in long-term care.

[54] Petersen P. (2012). Autonomy and empowerment of advanced practice nurses in New Mexico.

[55] Noser PS. (2011). Autonomous nurse practitioner practice: A position paper andaction plan for change.

[56] Cole C, Wellard S, Mummery J (2014). Problematising autonomy and advocacy in nursing. Nurs. Ethics..

[57] Eklund W. (2010). Japan and its healthcare challenges and potential contribution of neonatal nurse practitioners. J Perinat. Neonatal Nurs..

[58] Ruelens-Trinkaus DM. (2017). The Meaning and Experiences Of Professional Autonomy In Novice Registered Nurses.

[59] Espinosa L, Young A, Symes L, Haile B, Walsh T (2010). ICU nurses' experiences in providing terminal care. Crit. Care Nurs. Q..

[60] Medeiros ACd, Pereira QLC, Siqueira HCHd, Cecagno D, Moraes CL (2010). Participative management in permanent health education: view of the nurses. Rev. Bras. Enferm..

[61] AllahBakhshian M, Alimohammadi N, Taleghani F, Nik AY, Abbasi S, Gholizadeh L (2017). Barriers to intensive care unit nurses' autonomy in Iran: A qualitative study. Nurs. Outlook..

[62] Pursio K, Kankkunen P, Sanner‐Stiehr E, Kvist T (2021). Professional autonomy in nursing: An integrative review. J. Nurs. Manag..

[63] Lachman VD. (2007). Moral courage: a virtue in need of development?. Medsurg Nurs..

[64] Sung M-H, Kim Y-A, Ha M-J (2011). The relationships of professional self-concept, professional autonomy and self-esteem to job satisfaction of clinical nurses. J. Korean Acad. Fundam. Nurs..

[65] McDonald R, Harrison S, Checkland K, Campbell SM, Roland M (2007). Impact of financial incentives on clinical autonomy and internal motivation in primary care: ethnographic study. BMJ..

[66] Baljoon RA, Banjar HE, Banakhar MA (2018). Nurses’ work motivation and the factors affecting It: A scoping review. Int. J. Nurs. Clin. Pract..

[67] Weston MJ. (2010). Strategies for enhancing autonomy and control over nursing practice. Online J. Issues Nurs..

[68] Ulrich C, Soeken K, Miller N (2003). Predictors of nurse practitioners’ autonomy: Effects of organizational, ethical, and market characteristics. J. Am. Acad. Nurse Pract..

[69] Hanssen I. (2004). An intercultural nursing perspective on autonomy. Nurs. Ethics..

[70] Salhani D, Coulter I (2009). The politics of interprofessional working and the struggle for professional autonomy in nursing. Soc. Sci. Med..

[71] Wade GH (1999). Professional nurse autonomy: concept analysis and application to nursing education. J. Adv. Nurs..

[72] Petersen PA, Way SM (2017). The role of physician oversight on advanced practice nurses’ professional autonomy and empowerment.

[73] Stewart J, Stansfield K, Tapp D (2004). Clinical nurses’ understanding of autonomy: Accomplishing patient goals through interdependent practice. J. Nurs. Adm..

[74] Nouri A, Jouybari L, Sanagou A (2017). Nurses’perception of factors influencing professional autonomy in nursing: a qualitative study. Nurs. Midwifery J..

[75] Castro EM, Van Regenmortel T, Sermeus W, Vanhaecht K (2019). Patients’ experiential knowledge and expertise in health care: A hybrid concept analysis. Soc. Theory Health.

[76] Schutzenhofer KK, Musser DB (1994). Nurse characteristics and professional autonomy. J. Nurs. Scholarsh..

[77] Amini K, Negarandeh R, Ramezani‐Badr F, Moosaeifard M, Fallah R (2015). Nurses’ autonomy level in teaching hospitals and its relationship with the underlying factors. Int. Nurs. Pract..

[78] Maylone MM, Ranieri L, Griffin MTQ, McNulty R, Fitzpatrick JJ (2011). Collaboration and autonomy: Perceptions among nurse practitioners. J. Am. Acad. Nurse Pract..

[79] Maharmeh M. (2017). Understanding critical care nurses’ autonomy in Jordan. Leadersh Health Serv..

[80] Kuwano N, Fukuda H, Murashima S (2016). Factors affecting professional autonomy of Japanese nurses caring for culturally and linguistically diverse patients in a hospital setting in Japan. J. Transcult. Nurs..

